# Eye-Tracking-Based Evaluation of Cognitive Style and Driving Task Effects on AR-HUD Navigation Interfaces

**DOI:** 10.3390/s26133980

**Published:** 2026-06-23

**Authors:** Jing Li, Xinyu Feng, Min Lin, Hua Zhang

**Affiliations:** 1 College of Furnishings and Industrial Design, Nanjing Forestry University, Nanjing 210037, China; fengxinyunjfu@njfu.edu.cn (X.F.); linmin@njfu.edu.cn (M.L.); 2784534015@njfu.edu.cn (H.Z.); 2 Jiangsu Co-Innovation Center of Efficient Processing and Utilization of Forest Resources, Nanjing Forestry University, Nanjing 210037, China

**Keywords:** AR-HUD, navigation design, cognitive style, driving task, eye tracking, behavioral performance

## Abstract

**Highlights:**

**What are the main findings?**

- Stimulus-driven driving tasks significantly increased reaction times and visual-search behavior compared with goal-directed tasks.
- World-fixed displays improved visual efficiency during lane-change tasks, whereas screen-fixed displays enhanced attentional capture in pedestrian-warning scenarios.
- Field-dependent drivers exhibited significantly larger pupil diameters, indicating higher cognitive workload despite comparable behavioral performance.

**What is the implication of the main findings?**

- Eye-tracking-based sensing can support AR-HUD systems that dynamically optimize interface presentation according to driver workload and task context.

**Abstract:**

As augmented reality head-up display (AR-HUD) becomes increasingly integrated into intelligent vehicles, inappropriate interface designs may increase drivers’ cognitive workload and delay hazard responses. This study investigates how cognitive style, driving task type, and AR-HUD navigation design jointly influence drivers’ behavioral performance and visual attention. A total of 55 participants were recruited and screened using the Group Embedded Figures Test, with 38 drivers finally selected for a 2 × 4 × 2 driving-simulation experiment comparing world-fixed (WF) and screen-fixed (SF) interfaces across goal-directed and stimulus-driven tasks. Reaction times and eye-tracking indicators were analyzed using generalized linear models. Results show that stimulus-driven tasks significantly increased reaction times, with rear-vehicle scenarios producing the longest responses (mean = 1.420). During lane-change tasks, WF displays significantly reduced fixation duration (*p* < 0.001) and fixation counts (*p* < 0.001), whereas SF displays improved attentional efficiency during pedestrian-warning tasks. In addition, field-dependent drivers exhibited significantly larger pupil diameters, indicating higher cognitive workload. These findings provide sensor-based evidence for AR-HUD systems that dynamically optimize interface presentation according to task context and workload conditions.

## 1. Introduction

Advanced driver assistance systems (ADAS) and augmented reality head-up display (AR-HUD) are increasingly integrated into intelligent vehicle interfaces to support real-time information acquisition during driving [1,2]. By overlaying navigation, warning, and driving-related information directly onto the driver’s forward field of view, AR-HUD can reduce gaze transition frequency and improve information accessibility [3]. However, as AR-HUD interfaces become increasingly information-dense, excessive visual information may impose additional attentional demands and cognitive workload on drivers, thereby affecting driving safety and interaction efficiency [4,5].

Prior studies in human–computer interaction have demonstrated that interface visual styles and icon representations significantly influence visual search efficiency, attentional allocation, and cognitive processing performance [6,7]. Under high cognitive load, individuals generally exhibit prolonged visual search time, increased fixation behaviors, and heightened cognitive workload [8]—a pattern that also extends to driving [9]. Excessive interface complexity may further intensify attentional competition between driving-related tasks and interface-related information processing, leading to delayed responses and reduced behavioral performance [4,10]. Therefore, understanding how task demands affect cognitive workload and visual attention during AR-HUD interaction has become an important issue in driving human factors research [11].

In addition to task-related factors, individual cognitive differences may also influence drivers’ behavioral performance in complex visual environments [12]. Cognitive style, particularly the distinction between field-independent and field-dependent individuals, has been widely used to explain differences in information processing, attentional allocation, and visual search strategies [13,14]. FI individuals are generally considered more capable of extracting target information from complex visual backgrounds, whereas FD individuals tend to rely more heavily on external visual cues and contextual information [15]. Previous research has suggested that cognitive style may affect interface interaction efficiency, attentional control, and visual search behavior in digital interfaces and driving-related tasks [16,17]. However, the extent to which cognitive style moderates drivers’ cognitive workload and visual attention during AR-HUD interaction under varying task demands remains insufficiently understood.

Eye-tracking measures have been widely adopted to evaluate drivers’ cognitive workload and attentional processes during interface interaction [18,19]. Indicators such as fixation duration, pupil diameter, and response time provide objective evidence regarding visual attention allocation and mental effort [20,21]. In particular, pupil dilation has been closely associated with cognitive effort and attentional engagement during cognitively demanding tasks [22]. These physiological and behavioral indicators offer valuable insights into the underlying mechanisms of driver behavioral performance in AR-HUD environments.

Although previous studies have separately investigated AR-HUD interface design, driving workload, and cognitive style, limited research has systematically examined how driving task demands and cognitive style jointly influence visual search performance, fixation behavior, and cognitive workload during AR-HUD interaction. Moreover, existing studies have primarily focused on interface usability or behavioral performance outcomes, while the attentional and cognitive mechanisms underlying AR-HUD interaction remain underexplored.

Therefore, this study investigates the effects of driving task demand and cognitive style on drivers’ behavioral performance in AR-HUD environments. A mixed-factor experimental design was employed to examine response time, fixation behavior, and pupil diameter across different driving-task conditions and cognitive-style groups. The findings aim to provide empirical evidence for understanding cognitive workload and attentional allocation during AR-HUD interaction and offer theoretical support for human-centered AR-HUD interface design.

## 2. Related Work and Research Questions

### 2.1. Cognitive Style and Its Implications for AR-HUD Information Processing

Witkin, an American psychologist, was the first to introduce the concept of field-based cognitive styles, which is one of cognitive style theory’s earliest and most significant branches. Field dependence (FD) and field independence (FI) are two categories into which this idea can be separated [23]. While FD is more vulnerable to overall contextual influences and is better at seeing holistic phenomena, it has trouble breaking down individual details; FI tends to extract partial information from complicated situations and excels at analytical, structured information processing [24,25,26].

A consistent finding in spatial cognition research is the performance differential linked to FD and FI styles. FI individuals typically excel at tasks requiring mental manipulation (e.g., mental rotation) or disembedding figures from complex backgrounds, whereas FD individuals show superior performance in contexts that leverage external cues or integrated landmarks [27,28,29]. This pattern extends decisively to emerging interactive technologies. Studies on virtual navigation and AR interfaces confirm that FI users navigate information-sparse or complex virtual environments more efficiently, while FD users benefit from structured guidance and salient environmental anchors [30,31]. Therefore, individual cognitive styles robustly predict spatial performance. This predictive validity necessitates that AR-HUD navigation designs be tailored to them, thereby optimizing usability and enhancing driving safety.

### 2.2. Divergent Cognitive Demands of Goal-Directed and Stimulus-Driven Driving Tasks

From a cognitive control perspective, driving tasks can be partitioned along a continuum anchored by two prototypical modes: goal-directed (endogenous) attention and stimulus-driven (exogenous) attention [32,33]. The fundamental mechanism that distinguishes these modes is the source of attentional selection. Goal-directed tasks—such as lane changes, turn maneuvers, or route checks—are initiated by the driver’s current intentions and rely on top-down schemas. They require sustained anticipatory monitoring, working memory updating (e.g., tracking the position of surrounding vehicles while planning a gap), and voluntary reorientation of gaze to task-relevant locations [34,35]. Consequently, their cognitive workload is characterized by executive demand: the need to maintain and manipulate a mental model of the traffic situation over time.

In contrast, stimulus-driven tasks—such as sudden braking events, passing pedestrians, or collision warnings—are triggered by abrupt, salient events in the environment. Here, attentional capture is bottom-up and largely automatic, mediated by subcortical and frontoparietal circuits that prioritize biologically or motivationally significant stimuli [36,37]. The cognitive workload associated with stimulus-driven events is not primarily executive but reactive and perceptual-urgent: the driver must rapidly disengage from the current focus, reorient to the hazard, and execute a time-critical action (e.g., braking or swerving) [38].

This mechanistic distinction has direct design implications for AR-HUD. For goal-directed tasks, the AR interface should reduce the cost of mental transformation between the display and the road. For example, world-fixed navigation arrows that remain anchored to the physical lane can support spatial updating without requiring the driver to mentally rotate or translate a screen-fixed symbol. This is precisely why Cheng et al. [39] observed longer hazard-zone fixations and faster detection with AR-HUD during nighttime goal-directed navigation: the spatially registered information reduced the need for visual scanning. For stimulus-driven tasks, however, the priority is capturing attention as quickly as possible, even at the cost of abruptness. Dynamic warning icons that move in peripheral vision [40] or color-changing alert boxes [41] leverage bottom-up salience to override the current attentional set. In this case, world-fixed anchoring may be less critical than transient, high-contrast, motion-based signals that exploit the exogenous attention pathway.

Importantly, real driving involves rapid switching between these two modes [42]. AR-HUDs that excel at supporting goal-directed navigation but produce false alarms or cluttered static symbols may interfere with stimulus-driven hazard detection. Thus, the interface should dynamically adapt its presentation format based on the current task context. Distinguishing between types of driving tasks is crucial for evaluating the effectiveness of AR-HUD design and its alignment with varying cognitive demands.

### 2.3. AR-HUD Navigation Interface Design: World-Fixed Versus Screen-Fixed

The two dominant AR-HUD navigation paradigms—world-fixed (WF) and screen-fixed (SF)—differ fundamentally in the spatial reference frame they offer to the driver. WF displays anchor virtual graphics (e.g., turn arrows, lane boundaries) to egocentric or allocentric coordinates in the physical environment, such that the symbol remains locked to a specific point on the road (e.g., the upcoming intersection). As the driver’s head or vehicle moves, the graphic moves accordingly to maintain this world-registration, effectively “painting” information onto the scene [39]. This paradigm supports direct spatial mapping: the driver does not need to mentally translate a screen-based symbol into a real-world location because the symbol already occupies that location in the visual field.

In contrast, SF displays present graphics in a display-centric reference frame—typically a fixed region of the HUD (e.g., a corner or the center of the combiner). The driver must perform a mental spatial transformation to align the static symbol (e.g., a left-pointing arrow) with the appropriate real-world direction. This transformation imposes a spatial translation cost that increases with scene complexity and the number of competing stimuli [43]. Consequently, the cognitive efficiency of SF displays depends heavily on symbol semantics (e.g., the arrow shape is culturally learned) and the driver’s ability to rapidly map abstract symbols to actions—a process that recruits working memory and executive control.

The literature, however, shows no universal superiority of WF over SF; instead, the advantage is task- and context-dependent. WF displays excellence when the driving task requires spatial awareness and path integration, especially in unfamiliar or landmark-sparse environments. Zhao et al. [44] demonstrated that WF significantly improves spatial knowledge acquisition when landmarks are absent because it provides a continuous, registered spatial cue that supports the formation of an allocentric cognitive map. Conversely, when highly salient landmarks are already present, SF can be sufficient and may even reduce visual clutter. More surprisingly, in complex urban environments demanding high situational awareness, SF graphics have been shown to produce shorter HUD fixations, more hazard-scanning time, and lower subjective workload [43]. The mechanism behind this counterintuitive finding is likely occlusion and attentional capture: a persistently world-fixed ribbon may overlap with critical road features (e.g., pedestrians, traffic lights) and inadvertently draw fixations away from unexpected hazards. SF symbols, being confined to a predictable area, can be more easily ignored when not relevant, allowing drivers to voluntarily prioritize the road. Therefore, the choice between WF and SF should be treated not as a binary design decision but as a parameter tailored to the specific demands of the driving task and environment.

### 2.4. Research Hypotheses

The preceding mechanistic analysis reveals three interacting factors that jointly determine AR-HUD cognitive efficiency: (1) individual cognitive style (FI vs. FD), which modulates the reliance on internal restructuring versus external anchoring; (2) driving task mode (goal-directed vs. stimulus-driven), which determines whether the dominant cognitive demand is executive/sustained or reactive/urgent; and (3) navigation display paradigm (WF vs. SF), which differs in the spatial reference frame offered and the associated transformation cost. However, existing studies have largely examined these factors in isolation, leaving two critical gaps. First, it is unknown whether cognitive style moderates the relative advantage of WF over SF differently for goal-directed versus stimulus-driven tasks. Second, the interaction among all three factors has never been empirically tested. This study, therefore, addresses the following hypotheses:

**H1.**  *Cognitive style, driving task type, and navigation design each have a main effect on cognitive performance (reaction time) and cognitive workload (pupil dilation, fixation parameters).*

**H2.**  *There are significant two-way and three-way interactions; in particular, the advantage of WF over SF for FD drivers will be larger during goal-directed tasks than during stimulus-driven tasks.*

To test the hypotheses, eye-tracking is employed as a physiological sensing method, recording fixation duration, fixation count, average pupil diameter, and task-evoked pupillary response as real-time indices of cognitive workload and attentional allocation. These sensor-derived measures are intended to inform the development of AR-HUD systems that can dynamically switch between display paradigms based on both task context and real-time workload sensing.

## 3. Methods

### 3.1. Experimental Design

**Figure 1:**
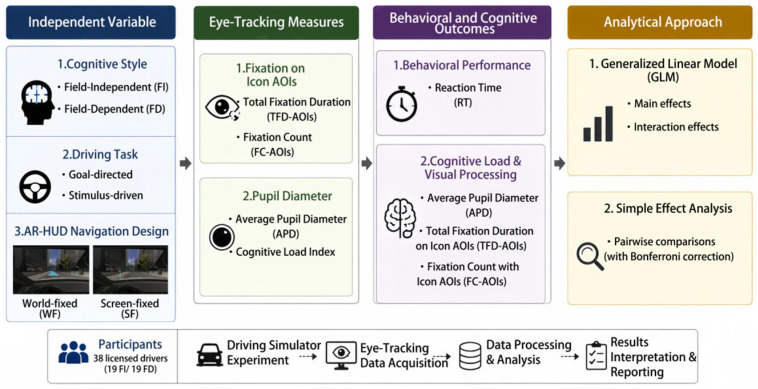
Conceptual model of the study.

The AR-HUD navigation design experiment employs a 2 × 2 × 4 mixed-factorial design: 2 (cognitive style: FD, FI) × 2 (navigation design: WF, SF) × 4 (driving task: 1. goal-directed: left turns, lane changes; 2. stimulus-driven: rear vehicles, passing pedestrians). Cognitive style served as a between-subjects factor. Navigation design and driving task were treated as within-subjects factors. The dependent variables included behavioral performance metrics (reaction time) and eye-tracking data (average pupil diameter, total fixation duration with icon AOIs, and fixation count with icon AOIs). To clarify the overall structure of this study, Figure 1 presents the conceptual model illustrating the relationships among cognitive style, driving task, AR-HUD navigation design, eye-tracking measures, and the research outcomes.

### 3.2. Participants

**Figure 2:**
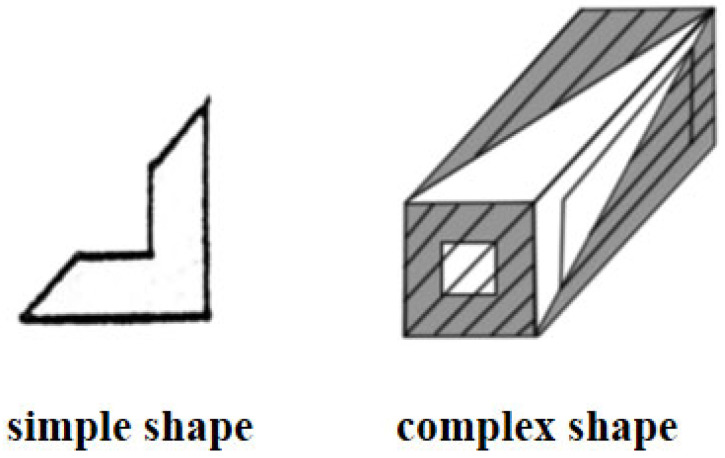
Representative examples of GEFT. GEFT was used to classify participants into FD and FI groups.

A total of 55 volunteers were recruited and screened with the Group Embedded Figures Test (GEFT) (Figure 2) to classify cognitive style [45,46]. Participants ranged from 20 to 30 years old (M = 23, SD = 2.1), comprising 20 males and 35 females. A total of 36% were social service professionals and 64% university students. All held valid driver’s licenses and had at least one year of driving experience. Their visual acuity and ocular symptoms were reviewed to ensure normal or corrected-to-normal vision. The absence of color vision deficiency was confirmed using a standard color vision test board. After providing written informed consent, each respondent completed the 20 min GEFT and received a ¥10 compensation.

To ensure statistical power, an a priori power analysis was conducted using G*Power 3.1 [47]. Based on the effect size (f = 0.25), an alpha level of 0.05, and a power of 0.95, the required sample size was calculated to be 32. The final valid sample of 38 participants surpassed this threshold, indicating adequate statistical power for the analysis. According to the theory underlying the GEFT, field-independent (FI) individuals are more adept at embedding simple figures from complex contexts, whereas FD individuals find this more difficult [27]. So, for classifying participants into FD and FI groups, we used a cut-off score at 11 correct items which has been used in practice [48]. Specifically, participants with a score of 11 or below were classified as FD, while those with a score above 11 were classified as FI. This criterion is consistent with the normative mean (11.4) established by Witkin et al. [23]. Applying this cutoff, 19 participants were classified as FD and 19 as FI. These 38 participants then proceeded to the main experiment, with their data included in the subsequent analyses.

### 3.3. Experimental Materials

As shown in Table 1, two driving tasks—left turns and lane changes—were created for the goal-directed task type, and two additional tasks—rear vehicles and passing pedestrians—were created for the stimulus-driven task type. This resulted in a total of four driving tasks. All experimental driving task scenarios were designed based on Li et al. [49]. To ensure standardization, the AR-HUD navigation was designed in two conditions: SF and WF. The SF icons remain fixed relative to the windshield plane, regardless of vehicle or environmental motion. In contrast, WF icons are anchored to real-world spatial positions, dynamically aligning with the road environment as the vehicle moves [43]. All icons were designed in compliance with national traffic sign standards to prevent ambiguity and ensure experimental validity.

### 3.4. Experimental Apparatus

**Figure 3:**
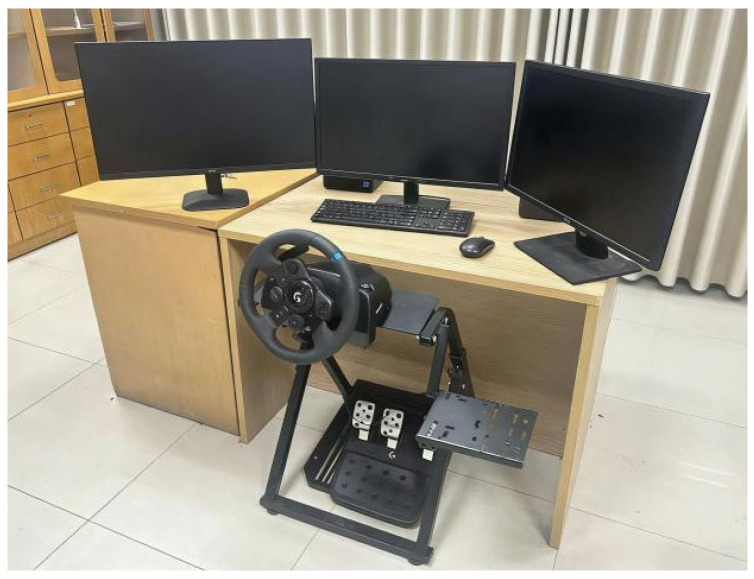
The experimental scene. The scene included a driving simulator, an eye-tracking system, and a triple-screen display environment.

This study used a Logitech G923 driving simulator (Logitech, Lausanne, Switzerland) with a steering wheel, brake pedal, and accelerator pedal. Simulation software made with the Unity 3D game engine (Unity version 2022.3.53) was used to generate the driving scenario. The fixed-base simulator lacks vestibular feedback, but the Unity 3D environment maintained a system latency below 66.6 ms [50]. The experiment used a Tobii TX300 integrated eye tracker (Tobii Technology AB, Danderyd, Sweden) with a triple-screen setup (total resolution 5760 × 1080) to track participants’ eye movements at a sampling rate of 300 Hz. The experiment was carried out in a sealed indoor space with a set light source intensity to guarantee constant ambient lighting conditions (maintaining goal illuminance at 300 ± 50 lux) (Figure 3).

### 3.5. Experimental Procedure

**Figure 4:**
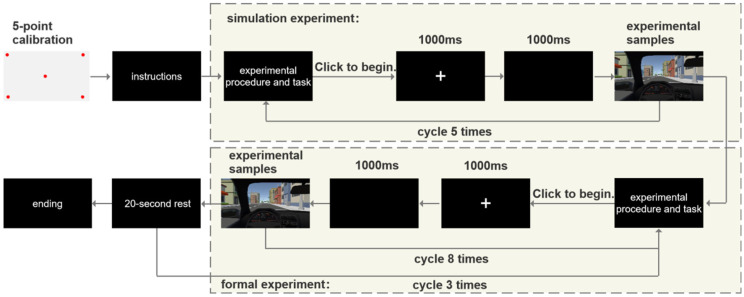
The experimental flow chart. The experiment consisted of calibration, practice trials, and formal trials under different AR-HUD navigation designs and driving tasks.

As illustrated in Figure 4, before the AR-HUD navigation design experiment began, the experimenter adjusted the driving-simulator seat for each participant and performed a five-point eye-tracker calibration to determine gaze position. After receiving the instructions, the participant familiarized themself with the procedure and tasks. Upon clicking “Start”, they completed five practice trials, followed by 24 formal trials (eight driving task scenarios × three repetitions). The practice session mirrored the formal sequence: a central “+” was shown for 1000 ms, followed by a 1000 ms blank screen and then one of the stimuli listed in Table 1. Participants responded to navigation cues by steering or activating the emergency brake. During the formal block, a 20 s rest period was provided after every eight randomly ordered trials. The experiment ended when all 24 trials had been completed.

**Figure 5:**
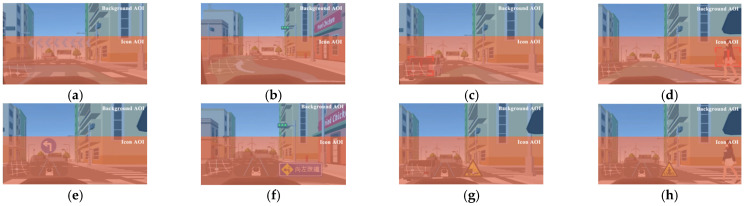
Definition of areas of interest (AOI) across driving scenarios: (**a**) WF—left turns; (**b**) WF—lane changes; (**c**) WF—rear vehicles; (**d**) WF—passing pedestrians; (**e**) SF—left turns; (**f**) SF—lane changes; (**g**) SF—rear vehicles; (**h**) SF—passing pedestrians.

During the experimental trials, the eye-tracking system systematically recorded participants’ eye movements and reaction times to quantify visual perception characteristics and identification efficiency. As illustrated in Figure 5, two distinct areas of interest (AOIs) were defined for the AR-HUD interface: the icon AOI and the background AOI.

### 3.6. Measures and Dependent Variables

Four dependent measures were used to assess drivers’ behavioral performance, visual attention allocation, and cognitive workload during AR-HUD interaction: reaction time (RT), total fixation duration within icon AOIs (TFD-AOIs), fixation count within icon AOIs (FC-AOIs), and average pupil diameter (APD). A summary of these is provided in Table 2.

## 4. Data Analysis

### 4.1. Data Processing

The experiment collected RT, TFD-AOIs, FC-AOIs and APD from 38 participants, which were analyzed using SPSS software (version 27). RT is an important parameter for determining a driver’ s cognitive workload and attention distribution. The research on AR-HUD for driving assistance used RT as a key metric [42]. Pupil size has been well examined and is thought to be a reliable indicator of cognitive workload or mental strain [51]. Ke J et al. [52] noted that eye movement metrics can effectively distinguish attention allocation patterns among users with different cognitive styles. RT served as an indicator of response accuracy, where negative values denoted premature responses (false alarms on target-absent trials) and latencies under 200 ms were treated as anticipatory guesses rather than genuine perceptual responses. To ensure analytical validity, missing or erroneous records were excluded during data cleaning, resulting in a final usable dataset comprising 93.3% of the original observations. The 6.7% exclusion rate (primarily due to eye-tracking data loss, blink interference, and RT shorter than 200 ms [53]) was similarly distributed across conditions: cognitive style (FD: 6.9%, FI: 6.5%), driving task (6.2–7.1%), and navigation design (WF: 6.4%, SF: 7.0%). Re-running the main Generalized Linear Model (GLM) after excluding the condition with the highest exclusion rate (rear vehicle, 7.1%) yielded identical significant effects, confirming that the minor variation did not bias conclusions. Tests confirmed that the collected experimental data constituted repeated measures and violated the assumption of normal distribution. Consequently, a GLM was applied to this cleaned data [54].

Cognitive efficiency was indexed by RT, TFD-AOIs and FC-AOIs. The APD indexed cognitive workload. To test these indices, we conducted a two-step analysis. First, a GLM examining three-way interactions among cognitive style, navigation design and driving task. Second, pairwise comparisons to evaluate condition-level differences in behavioral performance and eye-movement behavior. In addition to statistical significance testing, effect sizes were calculated to quantify the magnitude of observed differences. For post hoc pairwise comparisons, repeated-measures effect sizes (Cohen’s *d_z_*) were calculated based on the estimated marginal mean differences and standard errors following the recommendations of Lakens [55]. Cohen’s *d_z_* values were reported to quantify the magnitude of within-subject differences. Large Cohen’s *d_z_* values observed in some within-subject comparisons reflect the combination of small within-participant variability and consistent directional differences across repeated measurements, which may produce effect sizes exceeding conventional benchmarks.

### 4.2. Analysis of RT

The results of the GLM analysis of effects indicate that cognitive style, navigation design, the interaction between cognitive style and navigation design, the interaction between cognitive style and driving task, and the three-way interaction among all factors did not demonstrate statistically significant effects on RT (all pairwise comparisons *p* > 0.05). In contrast, driving task (χ^2^ = 223.420, *p* < 0.001) and the interaction between navigation design and driving task (χ^2^ = 34.573, *p* < 0.001) significantly influenced RT. Of note, contrary to our central hypothesis, the main effect of cognitive style, its interaction with navigation design, and the three-way interaction among cognitive style, driving task, and navigation design were all non-significant for RT (all *p* > 0.05). This absence of cognitive-style effects on response speed is addressed in Section 5.

The pairwise comparisons for driving tasks from GLM are presented in Table 3. RT differed significantly between every pair of driving tasks: left turns, lane changes, rear vehicles and passing pedestrians (all pairwise comparisons *p* < 0.05).

As shown in Table 4, in the left-turn and lane-change tasks, navigation designs differed significantly in RT (*p* < 0.05). In contrast, there was no statistically significant effect on RT for the tasks involving rear vehicles and passing pedestrians (*p* = 0.140, 0.709 > 0.05).

**Figure 6:**
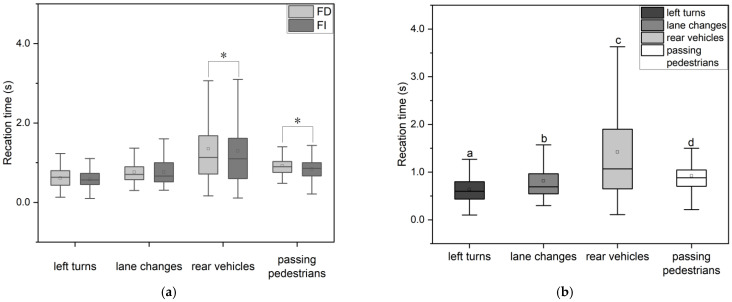
Descriptive comparison of RTs across driving tasks and cognitive styles. (**a**) RT patterns across cognitive styles under driving tasks, * *p* < 0.05. (**b**) Mean RT across driving tasks. Different letters indicate statistically significant differences between groups, while groups sharing the same letter are not significantly different from each other.

Although the omnibus GLM did not reveal a significant main effect of cognitive style or a significant Cognitive Style × Driving Task interaction, Figure 6a is presented to illustrate RT patterns across cognitive styles under different driving tasks. FD participants tended to exhibit longer RTs than FI participants in the two stimulus-driven tasks. In the rear-vehicle task, FD participants showed a longer RT (mean = 1.610) than FI participants (mean = 1.321). Similarly, in the passing-pedestrian task, FD participants again exhibited a longer RT (mean = 0.922) than FI participants (mean = 0.843). These observations are descriptive and should be interpreted cautiously given the absence of a significant omnibus interaction.

As shown in Figure 6b, RTs for the rear vehicles were the longest (mean = 1.420), significantly exceeding those for the passing pedestrians (mean = 0.926), lane changes (mean = 0.816), and left turns (mean = 0.636).

### 4.3. Analysis of TFD-AOIs

Regarding the TFD-AOIs, the GLM revealed that the two-way interactions between cognitive style and navigation design, the two-way interactions between cognitive style and driving task, as well as the three-way interaction among all factors, were not statistically significant (all pairwise comparisons *p* > 0.05). By contrast, the main effect of driving task (χ^2^ = 82.817, *p* < 0.001) and its interaction with navigation design (χ^2^ = 90.655, *p* < 0.001) were both significant.

**Figure 7:**
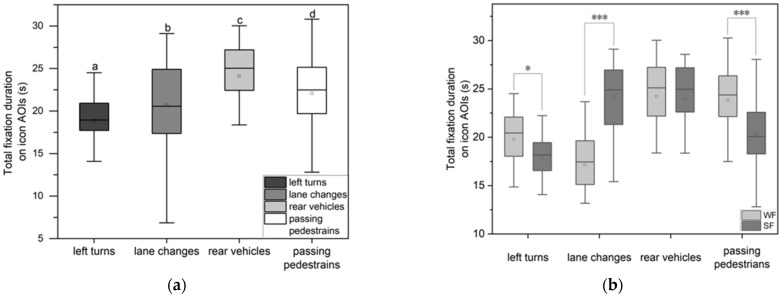
TFD-AOIs across driving task and navigation design. (**a**) Comparison of TFD-AOIs by different driving tasks. (**b**) Comparison of TFD-AOIs between navigation design and driving task. * *p* < 0.05. *** *p* < 0.001. Different letters indicate statistically significant differences between groups, while groups sharing the same letter are not significantly different from each other.

As shown in Table 3, pairwise comparisons (following the significant main effect of driving task) revealed that all pairs of driving tasks—left turns, lane changes, rear vehicles, and passing pedestrians—differed significantly in TFD-AOIs (all *p* < 0.05). As shown in Figure 7a, the TFD-AOIs for stimulus-driven tasks (rear vehicles and passing pedestrians) was significantly longer than for goal-directed tasks (left turns and lane changes). Among these, the rear vehicles task had the longest TFD-AOIs (mean = 24.098).

The GLM analysis (Table 4) showed that the influence of navigation design (WF vs. SF) on TFD-AOIs depended on the driving task. Significant differences between navigation designs were found for left turns, lane changes, and passing-pedestrians (all *p* < 0.01), but not for the rear vehicles task (*p* = 0.731 > 0.05). As shown in Figure 7b, during lane changes, WF resulted in shorter TFD-AOIs (mean = 17.178). In contrast, during passing pedestrian tasks, WF led to longer TFD-AOIs (mean = 23.816).

### 4.4. Analysis of FC-AOIs

The GLM analysis revealed that FC-AOIs were not significantly influenced by cognitive style, navigation design × cognitive style, driving task × cognitive style, or the three-way interaction (all *p* > 0.05). Conversely, significant main effects were found for navigation design (χ^2^ = 10.531, *p* < 0.05) and driving task (χ^2^ = 38.011, *p* < 0.001), as well as a significant interaction between navigation design and driving task (χ^2^ = 83.603, *p* < 0.001).

**Figure 8:**
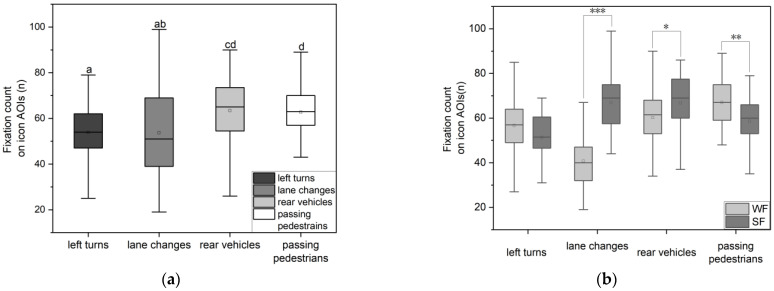
FC-AOIs across driving task and navigation design. (**a**) Comparison of FC-AOIs by different driving tasks. (**b**) Comparison of FC-AOIs between navigation design and driving task. * *p* < 0.05. ** *p* < 0.01, *** *p* < 0.001. Different letters indicate statistically significant differences between groups, while groups sharing the same letter are not significantly different from each other.

A GLM pairwise comparison was conducted for the driving task. As shown in Table 3, no significant differences in FC-AOIs were found between left turns and lane changes (*p* = 0.969 > 0.05) or between rear vehicles and passing-pedestrians (*p* = 0.704 > 0.05). However, FC-AOIs for both left turns and lane-change tasks were significantly lower than those for rear vehicles and passing pedestrian tasks (all *p* < 0.001). As shown in Figure 8a, while there was no significant difference in FC-AOIs between left turns (mean = 53.971) and lane changes (mean = 53.889), both were significantly lower than those for the rear vehicles (mean = 63.500) and passing pedestrians (mean = 62.694). The rear vehicles task recorded the highest FC-AOIs.

A GLM pairwise comparison was conducted to examine the interaction effect between navigation design and driving task on FC-AOIs. As shown in Table 4, significant differences between WF and SF displays were found for lane change, rear vehicles and passing-pedestrians (all *p* < 0.01). In contrast, no significant difference was observed for the left-turn task (*p* = 0.731 > 0.05). As shown in Figure 8b, during lane changes, WF resulted in shorter FC-AOIs (mean = 40.731). In contrast, during passing pedestrian tasks, WF led to longer FC-AOIs (mean = 66.833).

### 4.5. Analysis of APD

**Figure 9:**
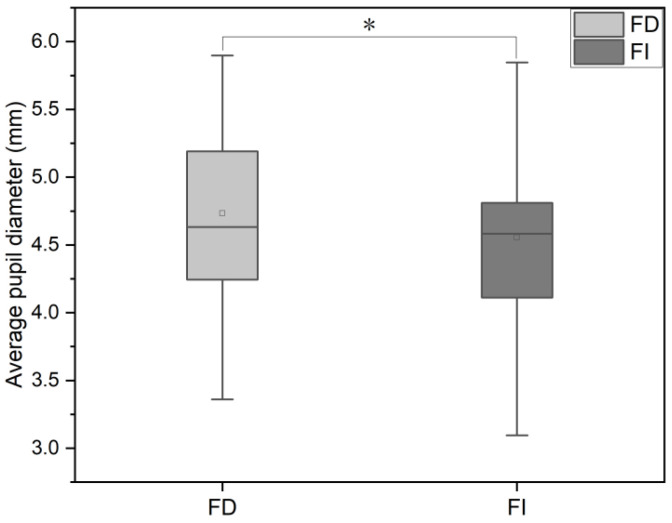
Comparison of APD by different cognitive styles. * *p* < 0.05.

The GLM analysis revealed no significant main effects or interactions on APD for navigation design, driving task, navigation design × cognitive style, driving task × cognitive style, or the three-way interaction (all *p* > 0.05). However, cognitive style exerted a significant main effect (χ^2^ = 5.521, *p* < 0.05). A significant main effect of cognitive style was observed for APD. As illustrated in Figure 9, the FD group (mean = 4.734) exhibited larger APD values than the FI group (mean = 4.556), indicating greater cognitive effort during task performance.

## 5. Discussion

### 5.1. Effects of Driving Task on Behavioral Performance and Visual Attention

#### 5.1.1. Driving Task as the Primary Determinant of Behavioral Performance and Visual Attention

The present findings provide strong support for H1 regarding the main effect of driving task type. Across the four dependent measures, driving task consistently exerted significant effects on RT, TFD-AOIs, and FC-AOIs. In general, stimulus-driven tasks generated longer RTs, longer fixation durations, and more frequent fixations than goal-directed tasks.

Among all task conditions, the rear vehicle scenario produced the longest RT, followed by the passing-pedestrian task, whereas left-turn and lane-change tasks resulted in substantially shorter response times. These findings suggest that stimulus-driven tasks impose greater attentional demands because drivers must rapidly detect and respond to unexpected external events. Unlike goal-directed tasks, which are supported by anticipatory attention and task planning, stimulus-driven tasks require abrupt attentional reorientation and rapid processing of peripheral visual information. The eye-tracking results further support this interpretation. Stimulus-driven tasks generated significantly longer TFD-AOIs and higher FC-AOIs than goal-directed tasks. This pattern indicates that drivers devoted more visual resources to processing AR-HUD information under stimulus-driven conditions. Previous studies have similarly reported that unexpected hazards increase visual search requirements and attentional workload during driving [56,57]. Sarkar et al. [56] demonstrated that peripheral hazard detection significantly prolongs response times, while Čulík et al. [58] reported slower reactions to unexpected events than to anticipated driving maneuvers.

The increased fixation duration and fixation counts observed in stimulus-driven tasks may reflect the need for continuous attentional updating when monitoring multiple sources of environmental information. Previous eye-tracking research has shown that unpredictable events increase both visual search activity and attentional demands [59,60]. Drivers must continuously evaluate target location, distance, and movement characteristics, resulting in greater visual workload [61]. Similar conclusions have been reported in intelligent transportation studies, where complex driving environments substantially increase attentional demands and cognitive resource allocation [62].

Taken together, these findings indicate that driving-task characteristics are the dominant factor influencing driver behavioral performance and visual attention during AR-HUD interaction.

#### 5.1.2. Cognitive Style Influences Cognitive Workload Rather than Behavioral Performance

The results only partially support H1 with respect to cognitive style. Although cognitive style did not significantly influence RT, TFD-AOIs, or FC-AOIs, a significant main effect was observed for APD.

Specifically, FD participants exhibited significantly larger pupil diameters than FI participants. Because APD is widely recognized as a physiological indicator of cognitive effort and mental workload [22], this finding suggests that FD drivers required greater cognitive resources to perform the same driving tasks. Importantly, this increased cognitive effort was not accompanied by poorer behavioral performance. RT differences between FD and FI participants were not statistically significant. This dissociation between cognitive workload and behavioral performance has been reported in previous neuroergonomics and eye-tracking studies [53,63]. Individual differences are often reflected more strongly in physiological measures than in overt behavioral outcomes when task demands remain within manageable limits. Under such circumstances, individuals may achieve comparable behavioral performance while relying on different levels of cognitive effort [64,65,66].

Several factors may explain the absence of significant behavioral differences. First, although the stimulus-driven tasks increased attentional demands, the overall complexity of the simulated driving environment may not have been sufficiently high for cognitive-style differences to emerge behaviorally. Second, the standardized and highly salient AR-HUD icons may have reduced the need for complex visual search strategies, thereby diminishing the influence of field dependence–independence. Third, the relatively homogeneous participant sample may have reduced between-subject variability.

Therefore, the present findings suggest that cognitive style primarily influences mental effort rather than observable behavioral performance under the current AR-HUD driving conditions.

#### 5.1.3. Navigation Design Influences Visual Search Efficiency

The results also partially support H1 regarding navigation design. Navigation design did not significantly influence RT or APD. However, significant effects were observed for eye-tracking measures, particularly TFD-AOIs and FC-AOIs.

These findings suggest that navigation design primarily affects how visual attention is allocated during AR-HUD interaction rather than directly influencing response speed or cognitive workload. The observed effects indicate that different display paradigms may alter the efficiency of visual search processes and information extraction, even when overall behavioral performance remains unchanged.

Consequently, the influence of navigation design appears to be task-dependent and is better reflected in eye-movement behavior than in global performance measures.

### 5.2. Absence of Cognitive-Style Interaction Effects

Contrary to H2, the expected interaction among cognitive style, driving task, and navigation design was not supported. Across all dependent measures, neither the Cognitive Style × Navigation Design interaction, the Cognitive Style × Driving Task interaction, nor the three-way Cognitive Style × Driving Task × Navigation Design interaction reached statistical significance. In particular, the hypothesized superiority of WF displays for FD drivers during goal-directed tasks was not observed. Therefore, the present results do not provide empirical support for the assumption that AR-HUD display effectiveness systematically varies according to cognitive style.

Several explanations may account for these null findings. First, the cognitive demands imposed by the experimental scenarios may not have exceeded the threshold necessary for cognitive-style differences to manifest behaviorally. Previous studies have shown that FI advantages become more evident under highly complex or uncertain conditions [29,31]. Second, the salience and standardization of the AR-HUD icons may have reduced reliance on individual visual-processing strategies, thereby attenuating cognitive-style effects. Third, the relatively homogeneous sample composition may have limited the variability required to detect interaction effects. Importantly, these null findings contribute to a more balanced understanding of AR-HUD human factors. The absence of significant interactions suggests that cognitive style may not be a dominant determinant of interface effectiveness under typical AR-HUD conditions. Therefore, the present findings do not provide sufficient evidence to support cognitive-style-based interface adaptation in AR-HUD systems.

Although the omnibus analyses did not reveal significant cognitive-style interactions, exploratory condition-level comparisons suggested that FD participants tended to exhibit longer RT than FI participants in the stimulus-driven tasks. For example, FD participants showed longer RT than FI participants in both the rear-vehicle and passing-pedestrian conditions. However, these comparisons were conducted after non-significant omnibus interaction tests and should therefore be interpreted cautiously. Future studies with larger samples and pre-registered analytical procedures are needed to determine whether such trends represent genuine cognitive-style effects.

### 5.3. Task-Dependent Effects of AR-HUD Navigation Design

Although H2 was not supported, a significant interaction between driving task and navigation design was consistently observed across multiple eye-tracking measures. This finding suggests that the effectiveness of AR-HUD display formats depends primarily on task characteristics rather than on cognitive style differences.

For goal-directed tasks such as lane changes, the observed visual-attention pattern suggests that spatially registered information may facilitate the integration of navigation cues with roadway elements. Previous studies have argued that spatial alignment reduces the need for mental transformation between interface information and environmental locations [39,43]. Such spatial congruence may facilitate faster visual integration and reduce visual-search demands. In contrast, for passing-pedestrian tasks, the SF design resulted in shorter fixation durations and fewer fixation counts. Stimulus-driven tasks require rapid attentional capture and immediate hazard detection. Under these conditions, centrally positioned and visually salient SF displays may facilitate faster detection of critical information and support efficient attentional reorientation [67].

**Figure 10:**
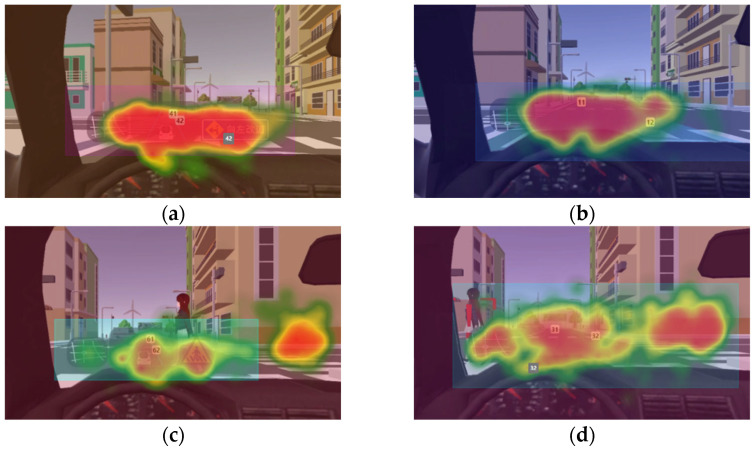
Eye-tracking heatmaps of AR-HUD navigation designs. (**a**) SF design in lane-change tasks; (**b**) WF design in lane-change tasks (**c**) SF design in passing pedestrians task; (**d**) WF design in passing pedestrians task.

To further illustrate these differences, eye-tracking heatmaps were examined (Figure 10). The heatmaps should be interpreted as qualitative visualizations that complement, rather than replace, the quantitative eye-tracking metrics. As shown in Figure 10a,b, WF displays in lane-change scenarios produced more concentrated fixation patterns around the task-relevant navigation area, whereas SF displays generated more dispersed visual attention. Conversely, in passing pedestrians scenarios (Figure 10c,d), SF displays produced more focused fixation distributions, while WF displays generated broader attentional dispersion across the interface and surrounding environment.

These findings suggest that no single display paradigm is universally optimal. Instead, AR-HUD effectiveness depends on the alignment between display characteristics and task requirements.

### 5.4. Limitations and Future Work

One limitation of this study is the reliance on reaction time and eye-tracking metrics (TFD-AOIs, FC-AOIs, and APD) as dependent measures. No direct driving-performance indicators—such as collision rate, lane-keeping deviation, braking intensity, or driving errors—were assessed. While these physiological and behavioral measures effectively capture attentional allocation and cognitive workload, they do not directly reflect driving safety or operational effectiveness. Therefore, the findings should be interpreted as reflecting differences in attentional allocation and cognitive processing efficiency rather than direct evidence of actual driving safety or driving performance. Future research should integrate complementary driving-performance metrics (e.g., vehicle control and error rates) alongside eye-tracking measures to provide a more comprehensive safety evaluation.

The study was conducted in a simulated driving environment, which offers experimental control and repeatability but cannot fully replicate the complexity of real-world traffic conditions. Real-world driving involves additional sources of uncertainty, including traffic density, environmental distractions, and situational pressure, which may affect drivers’ eye-movement patterns, visual search behavior, and cognitive workload. Therefore, the observed effects of AR-HUD navigation interfaces should be interpreted within the context of simulated driving. Future research should validate the findings through on-road or mixed-reality driving experiments.

The participant sample consisted primarily of young drivers with relatively similar educational backgrounds and driving experience, which may limit the generalizability of the results to other driver populations, such as elderly or novice drivers.

The exploratory comparisons between FD and FI participants should also be interpreted cautiously. As no significant omnibus interaction involving cognitive style was observed, these analyses were intended to identify potential trends rather than provide confirmatory evidence. Future studies with larger samples are needed to further examine cognitive-style effects.

Future research should incorporate objective driving-performance indicators to further validate the practical implications of different AR-HUD navigation designs, and may also include additional physiological measures, such as EEG or fNIRS, to provide a more comprehensive understanding of the cognitive mechanisms underlying AR-HUD interaction.

### 5.5. Design Implications

The current findings indicate that the effectiveness of AR-HUD depends more on the demands of the driving task than on stable differences in drivers’ cognitive styles.

For goal-directed tasks, such as lane changes, WF displays appear to facilitate more efficient visual processing by maintaining spatial consistency between virtual information and the roadway environment. For stimulus-driven tasks, such as pedestrian warnings, SF displays may improve attentional capture and support faster hazard detection.

Rather than supporting a universal display strategy or a cognitive-style-specific adaptation framework, the results indicate that navigation-display effectiveness depends primarily on task context. Therefore, future AR-HUD design should prioritize task-aware interface strategies that align display characteristics with the cognitive demands of different driving situations. Although cognitive style influenced cognitive workload, the present findings do not provide sufficient evidence to support cognitive-style-specific interface adaptation. Instead, task context appears to be a more reliable determinant of AR-HUD effectiveness.

## 6. Conclusions

This study investigated the effects of cognitive style, driving task, and AR-HUD navigation design on driver behavior, visual attention, and cognitive workload in a simulated driving environment.

The findings indicate that driving-task characteristics play a more important role than cognitive-style differences in shaping driver responses during AR-HUD interaction. While cognitive style was associated with differences in cognitive workload, its influence on behavioral performance was limited under the experimental conditions. In contrast, the effectiveness of AR-HUD navigation displays varied across driving tasks, highlighting the importance of considering task requirements when evaluating interface performance.

From a design perspective, the results suggest that AR-HUD interfaces should be developed with greater attention to task context rather than relying on a single display strategy. Matching display characteristics with the demands of specific driving situations may provide a more effective approach for supporting driver attention and information processing.

The present study provides empirical evidence for task-oriented AR-HUD design and highlights the importance of considering task context when evaluating navigation interfaces. The findings should be interpreted within the scope of the measures employed in this study and provide evidence regarding drivers’ visual attention and cognitive processing under different AR-HUD navigation designs and driving tasks. Future research should further validate these findings using more diverse driver populations, additional driving-performance indicators, and real-world driving environments.

## Figures and Tables

**Table 1:** Experimental stimulus materials. Driving tasks include goal-directed (left turns, lane changes) and stimulus-driven (rear vehicles, passing pedestrians) conditions under WF and SF navigation designs.

Driving Task Type	Driving Task	Navigation Design	
		World-Fixed (WF)	Screen-Fixed (SF)
goal-directed	left turns	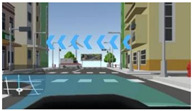	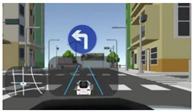
	lane changes	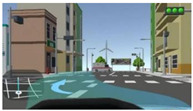	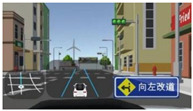
stimulus-driven	rear vehicles	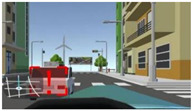	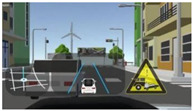
	passing pedestrians	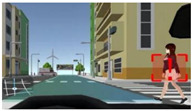	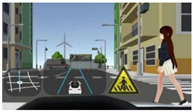

**Table 2:** Description of dependent measures and their interpretations.

Measure	Definition	Rationale	Interpretation
RT	reaction time	behavioral performance	lower values indicate faster responses
TFD-AOIs	total fixation duration within icon AOIs	visual attention allocation	higher values indicate greater attention demand
FC-AOIs	fixation count with icon AOIs	visual search behavior	higher values indicate increased visual search
APD	average pupil diameter	cognitive workload	higher values indicate greater mental effort

**Table 3:** Pairwise comparisons of driving task effects on RT, TFD-AOIs and FC-AOIs. a: The significance level is 0.05.

Measure	Driving Task (I–J)	Mean Difference (I–J)	Standard Error	*p*	Lower 95% CL of Mean	Upper 95% CL of Mean	Cohen’ s *d_z_*
RT	left turns—lane changes	−0.180 ^a^	0.054	0.001	−0.285	−0.075	−0.545
	left turns—rear vehicles	−0.784 ^a^	0.055	0.000	−0.891	−0.677	−2.326
	left turns—passing pedestrians	−0.290 ^a^	0.054	0.000	−0.395	−0.184	−0.872
	lane changes—rear vehicles	−0.604 ^a^	0.055	0.000	−0.711	−0.497	−1.791
	lane changes—passing pedestrians	−0.110 ^a^	0.054	0.041	−0.215	−0.004	−0.331
	rear vehicles—passing pedestrians	0.494 ^a^	0.055	0.000	0.387	0.602	1.460
TFD-AOIs	left turns—lane changes	−1.8558 ^a^	0.602	0.002	−3.036	−0.676	−0.500
	left turns—rear vehicles	−5.256 ^a^	0.598	0.000	−6.427	−4.084	−1.427
	left turns—passing pedestrians	−3.239 ^a^	0.598	0.000	−4.410	−2.067	−0.879
	lane changes—rear vehicles	−3.400 ^a^	0.596	0.000	−4.567	−2.233	−0.926
	lane changes—passing pedestrians	−1.383 ^a^	0.596	0.020	−2.550	−0.216	−0.377
	rear vehicles—passing pedestrians	2.017 ^a^	0.591	0.001	0.858	3.176	0.553
FC-AOIs	left turns—lane changes	0.080	2.098	0.969	−4.030	4.190	0.006
	left turns—rear vehicles	−9.530 ^a^	2.105	0.000	−13.660	−5.400	−0.734
	left turns—passing pedestrians	−8.720 ^a^	2.127	0.000	−12.890	−4.550	−0.665
	lane changes—rear vehicles	−9.610 ^a^	2.091	0.000	−13.710	−5.510	−0.746
	lane changes—passing pedestrians	−8.800 ^a^	2.113	0.000	−12.950	−4.660	−0.676
	rear vehicles—passing pedestrians	0.810	2.121	0.704	−3.350	4.960	0.062

**Table 4:** Pairwise comparisons of the interaction effect between navigation design and driving task on RT, TFD-AOIs and FC-AOIs. a: The significance level is 0.05.

Measure	Driving Task	Comparison Between Navigation Design (I–J)	Mean Difference (I–J)	Standard Error	*p*	Lower 95% CL of Mean	Upper 95% CL of Mean	Cohen’ s *d_z_*
RT	left turns	WF-SF	0.260 ^a^	0.076	0.001	0.111	0.409	0.555
	lane changes	WF-SF	−0.364 ^a^	0.076	0.000	−0.513	−0.216	−0.780
	rear vehicles	WF-SF	−0.116	0.079	0.140	−0.271	0.038	−0.240
	passing pedestrians	WF-SF	−0.029	0.076	0.709	−0.178	0.121	−0.061
TFD-AOIs	left turns	WF-SF	1.910 ^a^	0.854	0.025	0.236	3.584	0.363
	lane changes	WF-SF	−7.040 ^a^	0.848	0.000	−8.702	−5.377	−1.347
	rear vehicles	WF-SF	0.288	0.836	0.731	−1.351	1.926	0.056
	passing pedestrians	WF-SF	3.471 ^a^	0.836	0.000	1.832	5.109	0.673
FC-AOIs	left turns	WF-SF	5.240	2.987	0.079	−0.610	11.100	0.285
	lane changes	WF-SF	−26.320 ^a^	2.946	0.000	−32.090	−20.540	−1.449
	rear vehicles	WF-SF	−6.560 ^a^	2.968	0.027	−12.380	−0.750	−0.359
	passing pedestrians	WF-SF	8.280 ^a^	3.030	0.006	2.340	14.220	0.443

## Data Availability

Data are contained within the article.

## Abbreviations

AR-HUD	Augmented reality head-up display
WF	World-fixed
SF	Screen-fixed
RT	Reaction Time
APD	Average pupil diameter
TFD-AOIs	Total fixation duration within icon AOIs
FC-AOIs	Fixation count within icon AOIs
GLM	Generalized Linear Model

## References

[1] Kim H., Gabbard J.L. (2022). Assessing distraction potential of augmented reality head-up displays for vehicle drivers. Hum. Factors.

[2] Winkler M., Soleimani M. (2025). A review of augmented reality heads up display in vehicles: Effectiveness, application, and safety. Int. J. Hum.–Comput. Interact..

[3] Ma X., Jia M., Hong Z., Kwok A.P.K., Yan M. (2021). Does augmented-reality head-up display help? A preliminary study on driving performance through a VR-simulated eye movement analysis. IEEE Access.

[4] Wickens C.D. (2008). Multiple resources and mental workload. Hum. Factors.

[5] Horrey W.J., Wickens C.D. (2007). In-vehicle glance duration: Distributions, tails, and model of crash risk. Transp. Res. Rec..

[6] Shen Z., Chen T., Wang Y., Li M., Chen J., Hu Z. (2024). Skeuomorphic or flat? The effects of icon style on visual search and recognition performance. Displays.

[7] Liang Y., Lee J.D., Yekhshatyan L. (2012). How dangerous is looking away from the road? Algorithms predict crash risk from glance patterns in naturalistic driving. Hum. Factors.

[8] Bai Y., Shao J., Zhang Y., Chen L., Zhao X., Tian F., Xue C. (2022). ERP study of mine management system warning interface under fatigue. Int. J. Environ. Res. Public Health.

[9] Han L., Du Z., Wang S. (2025). Assessment of drivers’ visual search patterns and cognitive load during driving in curved tunnels. Traffic Inj. Prev..

[10] Lavie N. (2005). Distracted and confused? Selective attention under load. Trends Cogn. Sci..

[11] Endsley M.R. (2017). From here to autonomy: Lessons learned from human–automation research. Hum. Factors.

[12] Riding R., Cheema I. (1991). Cognitive styles—An overview and integration. Educ. Psychol..

[13] Witkin H.A., Moore C.A., Goodenough D.R., Cox P.W. (1977). Field-dependent and field-independent cognitive styles and their educational implications. Rev. Educ. Res..

[14] Zhang L. (2004). Revisiting the predictive power of thinking styles for academic performance. J. Psychol..

[15] Tinajero C., Páramo M.F. (1997). Field dependence-independence and academic achievement: A re-examination of their relationship. Br. J. Educ. Psychol..

[16] Chen S.Y., Macredie R.D. (2002). Cognitive styles and hypermedia navigation: Development of a learning model. J. Am. Soc. Inf. Sci. Technol..

[17] Zivi P., Giancola M., Nori R., Piccardi L., D’AMico S., Palmiero M. (2025). Field dependent-independent cognitive style as a predictor of malevolent creativity: A multifaceted approach. Front. Psychol..

[18] Holmqvist K., Nyström M., Andersson R., Dewhurst R., Jarodzka H., Van de Weijer J. (2011). Eye Tracking: A Comprehensive Guide to Methods and Measures.

[19] Duchowski A.T., Duchowski A.T. (2017). Eye Tracking Methodology: Theory and Practice.

[20] Just M.A., Carpenter P.A. (1976). Eye fixations and cognitive processes. Cogn. Psychol..

[21] Beatty J. (1982). Task-evoked pupillary responses, processing load, and the structure of processing resources. Psychol. Bull..

[22] Claron J., Royo J., Arcizet F., Deffieux T., Tanter M., Pouget P. (2022). Covariations between pupil diameter and supplementary eye field activity suggest a role in cognitive effort implementation. PLoS Biol..

[23] Witkin H.A., Goodenough D.R. (1977). Field dependence and interpersonal behavior. Psychol. Bull..

[24] Kozhevnikov M. (2007). Cognitive styles in the context of modern psychology: Toward an integrated framework of cognitive style. Psychol. Bull..

[25] Wang M., Ji X., Gan J., Guo Y. (2024). Differences in time-based prospective memory between field-independent and field-dependent cognitive styles under different time monitoring conditions. S. Afr. J. Psychol..

[26] Mu D., Zou M., Chen Y. (2025). How do different cognitive styles learners deal with the bullet screen interruption in instructional videos? An eye-tracking study. BMC Psychol..

[27] Zhu Y., Gu J., Lin Y., Chen M., Guo Q., Du X., Xue C. (2022). Field Cognitive Styles on Visual Cognition in the Event Structure Design of Bivariate Interactive Dorling Cartogram—The Similarities and Differences of Field-Independent and Field-Dependent Users. ISPRS Int. J. Geo-Inf..

[28] Park H., Dixit M.K., Pariafsai F. (2024). Towards personally relevant navigation: The differential effects of cognitive style and map orientation on spatial knowledge development. Appl. Sci..

[29] Li H., Zhang Y., Wu C., Mei D. (2016). Effects of field dependence-independence and frame of reference on navigation performance using multi-dimensional electronic maps. Personal. Individ. Differ..

[30] Cai J.-Y., Wang R.-F., Wang C.-Y., Ye X.-D., Li X.-Z. (2021). The influence of learners’ cognitive style and testing environment supported by virtual reality on English-speaking learning achievement. Sustainability.

[31] Nori R., Boccia M., Palmiero M., Piccardi L. (2023). The contribution of field independence in virtual spatial updating. Curr. Psychol..

[32] Miller E.K., Cohen J.D. (2001). An integrative theory of prefrontal cortex function. Annu. Rev. Neurosci..

[33] Theeuwes J. (2025). Attentional capture and control. Annu. Rev. Psychol..

[34] Frissen I., Mars F. (2024). Planning lane changes using advance visual and haptic information. Psychol. Res..

[35] Noonan T.Z., Gershon P., Mehler B., Reimer B. (2023). Visual attention patterns in automated and manual lane change maneuvers. Proceedings of the Human Factors and Ergonomics Society Annual Meeting.

[36] Innes R., Todd J. (2022). Modeling distraction: How stimulus-driven attention capture influences goal-directed behavior. J. Cogn. Neurosci..

[37] Trende A., Unni A., Jablonski M., Biebl B., Lüdtke A., Fränzle M., Rieger J.W. (2022). Driver’s turning intent recognition model based on brain activation and contextual information. Front. Neuroergon..

[38] Horswill M.S. (2016). Hazard perception in driving. Curr. Dir. Psychol. Sci..

[39] Cheng Y., Zhong X., Tian L. (2023). Does the AR-HUD system affect driving behaviour? An eye-tracking experiment study. Transp. Res. Interdiscip. Perspect..

[40] Wu Z., Liang Y., Liu G., Ai X. (2024). Comparative analysis of ar-huds crash warning icon designs: An eye-tracking study using 360° panoramic driving simulation. Sustainability.

[41] Wang J., Yang J., Fu Q., Zhang J., Zhang J. (2024). A new dynamic spatial information design framework for AR-HUD to evoke drivers’ instinctive responses and improve accident prevention. Int. J. Hum.-Comput. Stud..

[42] Merenda C., Kim H., Tanous K., Gabbard J.L., Feichtl B., Misu T., Suga C. (2018). Augmented reality interface design approaches for goal-directed and stimulus-driven driving tasks. IEEE Trans. Vis. Comput. Graph..

[43] Smith M., Gabbard J.L., Burnett G., Hare C., Singh H., Skrypchuk L. (2021). Determining the impact of augmented reality graphic spatial location and motion on driver behaviors. Appl. Ergon..

[44] Zhao Y., Stefanucci J., Creem-Regehr S., Bodenheimer B. (2023). Evaluating augmented reality landmark cues and frame of reference displays with virtual reality. IEEE Trans. Vis. Comput. Graph..

[45] Emmett D., Clifford B.R., Gwyer P. (2003). An investigation of the interaction between cognitive style and context reinstatement on the memory performance of eyewitnesses. Personal. Individ. Differ..

[46] Sharma D., Sharma M., Kaur P., Awasthy S., Kaushal S., D’SOuza M., Bagler G., Modi S. (2023). Camouflage detection and its association with cognitive style: A functional connectivity study. Brain Connect..

[47] Zhang N., Zhang J., Jiang S., Di X., Li W. (2024). Moderating Effects of Visual Order in Graphical Symbol Complexity: The Practical Implications for Design. Appl. Sci..

[48] Hong J.-C., Hwang M.-Y., Tam K.-P., Lai Y.-H., Liu L.-C. (2012). Effects of cognitive style on digital jigsaw puzzle performance: A GridWare analysis. Comput. Hum. Behav..

[49] Li R., Chen Y.V., Zhang L., Shen Z., Qian Z.C. (2020). Effects of perception of head-up display on the driving safety of experienced and inexperienced drivers. Displays.

[50] Xiong H., Wang Z., Wu G., Pan Y. (2022). Design and implementation of digital twin-assisted simulation method for autonomous vehicle in car-following scenario. J. Sens..

[51] Bruya B., Tang Y.Y. (2018). Is attention really effort? Revisiting Daniel Kahneman’s influential 1973 book attention and effort. Front. Psychol..

[52] Ke J., Liao P., Li J., Luo X. (2023). Effect of information load and cognitive style on cognitive load of visualized dashboards for construction-related activities. Autom. Constr..

[53] Wu Y., Zhang Y., Zheng B. (2024). Workload assessment of operators: Correlation between NASA-TLX and pupillary responses. Appl. Sci..

[54] Pekár S., Brabec M. (2018). Generalized estimating equations: A pragmatic and flexible approach to the marginal GLM modelling of correlated data in the behavioural sciences. Ethology.

[55] Lakens D. (2013). Calculating and reporting effect sizes to facilitate cumulative science: A practical primer for t-tests and ANOVAs. Front. Psychol..

[56] Sarkar A., Alambeigi H., McDonald A., Markkula G., Hickman J. (2021). Role of peripheral vision in brake reaction time during safety critical events. Proceedings of the Human Factors and Ergonomics Society Annual Meeting.

[57] Jurecki R.S., Stańczyk T.L. (2014). Driver reaction time to lateral entering pedestrian in a simulated crash traffic situation. Transp. Res. Part F Traffic Psychol. Behav..

[58] Čulík K., Kalašová A., Štefancová V. (2022). Evaluation of driver’s reaction time measured in driving simulator. Sensors.

[59] Ktistakis E., Skaramagkas V., Manousos D., Tachos N.S., Tripoliti E., Fotiadis D.I., Tsiknakis M. (2022). COLET: A dataset for COgnitive workLoad estimation based on eye-tracking. Comput. Methods Programs Biomed..

[60] Kimura M., Kimura K., Takeda Y. (2022). Assessment of driver’s attentional resource allocation to visual, cognitive, and action processing by brain and eye signals. Transp. Res. Part F Traffic Psychol. Behav..

[61] Zhang Y., Jiang P., Wang S., Cheng S., Xu J., Liu Y. (2024). Study on the Driver Visual Workload in High-Density Interchange-Merging Areas Based on a Field Driving Test. Sensors.

[62] Nagy V., Kovács G., Földesi P., Kurhan D., Sysyn M., Szalai S., Fischer S. (2023). Testing road vehicle user interfaces concerning the driver’s cognitive load. Infrastructures.

[63] Fairclough S.H., Mulder L.J.M. (2012). Psychophysiological processes of mental effort investment. How Motivation Affects Cardiovascular Response: Mechanisms and Applications.

[64] Ma J., Li Y., Zuo Y. (2024). Design and evaluation of ecological interface of driving warning system based on AR-HUD. Sensors.

[65] Beatty J., Lucero-Wagoner B. (2000). The pupillary system. Handbook of Psychophysiology.

[66] Van der Wel P., Van Steenbergen H. (2018). Pupil dilation as an index of effort in cognitive control tasks: A review. Psychon. Bull. Rev..

[67] Rusch M.L., Schall M.C., Gavin P., Lee J.D., Dawson J.D., Vecera S., Rizzo M. (2013). Directing driver attention with augmented reality cues. Transp. Res. Part F Traffic Psychol. Behav..

